# Stereotaxic gamma knife surgery in treatment of critically located pilocytic astrocytoma: preliminary result

**DOI:** 10.1186/1477-7819-5-39

**Published:** 2007-03-29

**Authors:** Raef FA Hafez

**Affiliations:** 1 International Medical Center, Gamma Knife Center, Cairo- Egypt

## Abstract

**Background:**

Low-grade gliomas are uncommon primary brain tumors, located more often in the posterior fossa, optic pathway, and brain stem and less commonly in the cerebral hemispheres.

**Case presentations:**

Two patients with diagnosed recurrent cystic pilocytic astrocytoma critically located within the brain (thalamic and brain stem) were treated with gamma knife surgery. Gamma knife surgery (GKS) did improve the patient's clinical condition very much which remained stable later on. Progressive reduction on the magnetic resonance imaging (MRI) studies of the solid part of the tumor and almost disappearance of the cystic component was achieved within the follow-up period of 36 months in the first case with the (thalamic located lesion) and 22 months in the second case with the (brain stem located lesion).

**Conclusion:**

Gamma knife surgery represents an alternate tool in the treatment of recurrent and/or small postoperative residual pilocytic astrocytoma especially if they are critically located

## Background

Low-grade gliomas are uncommon primary brain tumors classified as histological grades I or II in the World Health Organization (WHO) classification. The most common variants are pilocytic astrocytoma, low-grade astrocytomas, oligodendrogliomas, and mixed oligo-astrocytomas located more often in the posterior fossa, optic pathway, and brain stem and less commonly in the cerebral hemispheres. Prognostic factors that predict progression-free and overall survival include young age, pilocytic histology, good Karnofsky performance status, gross total resection, lack of enhancement on imaging, and small preoperative tumor volumes. Edema and vasogenic effects are typically managed with corticosteroids. The rationale for open craniotomy depends on the need for immediate palliation of symptoms by reduction of intracranial pressure or focal mass effect, and/or improved oncologic control. [1,2]

Gross total resection of low-grade glioma is generally defined as the absence of residual enhancement on contrast-enhanced postoperative MRI scan. Most retrospective studies suggest that patients who have undergone a gross total resection of tumor have improved survival. Depending upon the proximity of the tumor to eloquent brain areas, gross total resection may or may not be possible. In these cases a stereotactic biopsy is required to provide the histological diagnosis. Adjuvant radiotherapy is recommended for patients with incompletely resected grade II tumors or for patients older than age 40 regardless of extent of resection, it may be considered for any pilocytic astrocytoma from which a biopsy has been made. Radiosurgery and/or experimental chemotherapy may provide some measure of local control in the recurrent disease setting [3-6]

Stereotactic radiotherapy provides excellent local control for small, localized low-grade glial tumors. Marginal failures have not been observed, supporting the use of limited margins radiation dose to minimize late sequelae using stereotactic leksell gamma knife technique [7,8]

## Case presentations

### Case 1

A 10 years old girl presented with right side weakness, with vomiting attacks and blurring of vision. MRI delineated a large cystic lesion at left thalamic region with solid part. Stereotactic biopsy from the solid portion and aspiration of the cyst revealed pilocytic astrocytoma. Post aspiration the child improved clinically, but three months later the symptoms recurred and MRI revealed recurrence of the cystic part of the tumor that was treated again with stereotactic aspiration of the fluid, followed with stationary course. There was no history of patient receiving radiotherapy.

The child was referred to our Gamma Knife Center in October 2003; 6 months post second stereotactic aspiration with right side weakness more in the upper limb, with right facial palsy, and headache. MRI revealed large nonenhanced left thalamic cystic lesion with small two hyperintense intracystic solid parts.

**Figure 1:**
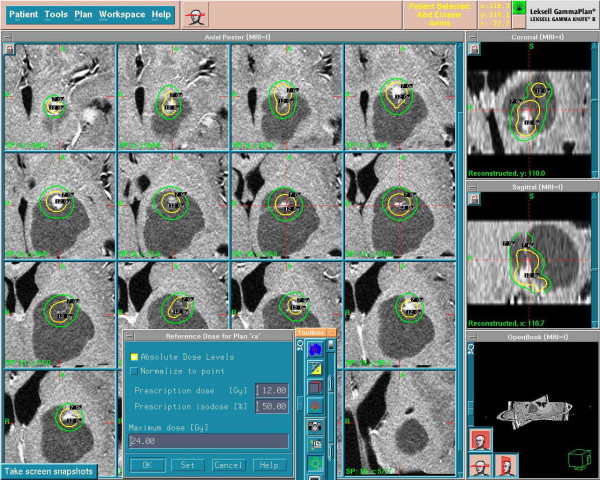
Gamma Knife treatment planning for the solid parts of the recurrent cystic pilocytic astrocytoma.

**Figure 2:**
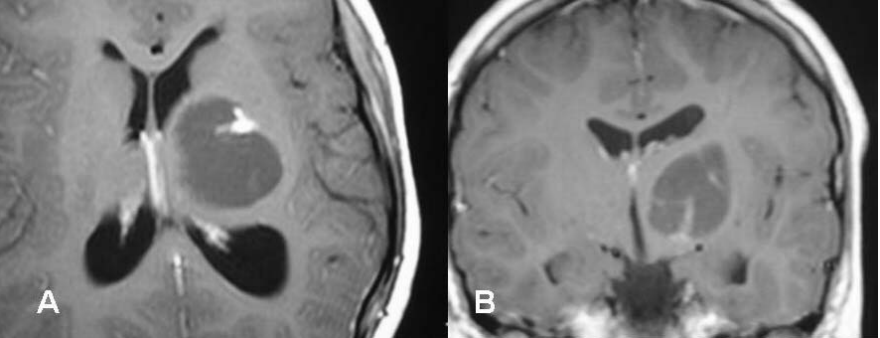
MRI brain showed left thalamic postoperative recurrent cystic pilocytic astrocytoma (solid and cystic part), before Gamma Knife surgery.

**Figure 3:**
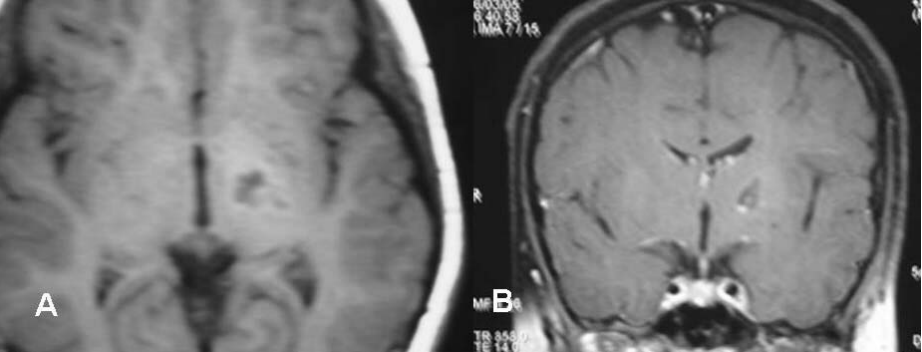
MRI brain follow-up after 30 months of Gamma Knife surgery of left thalamic recurrent pilocytic astrocytoma with marked reduction of the lesion size.

The child was treated with Gamma Knife with peripheral prescription dose of 12 Gy to the volume of the solid parts with maximum dose of 24 Gy at 50% isodose curve (figure 1&2). The first follow-up after GKS was at 6 months where no change of the volume of the lesion was seen however there was improvement of the clinical condition. At one year there was marked improvement in clinical evaluation with no weakness and no symptoms of increased intracranial pressure, MRI brain revealed reduction in tumor size as well as loss of the solid portion contrast enhancement (Figure 3).

In October 2006, after 36months of follow-up, MRI of the brain showed even more reduction in tumor and no solid part enhancement was seen with good clinical stable condition.

### Case 2

**Figure 4:**
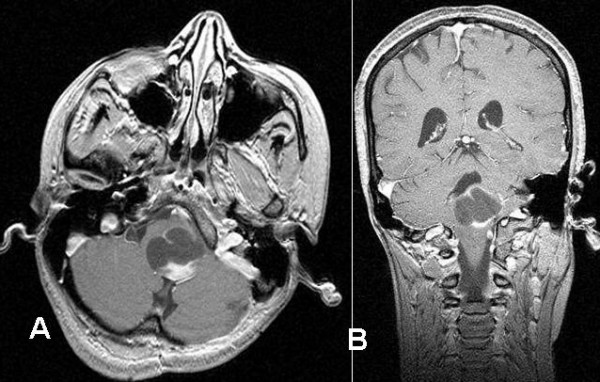
Stereotactic MRI brain showed recurrent postoperative brain stem cystic pilocytic astrocytoma.

**Figure 5:**
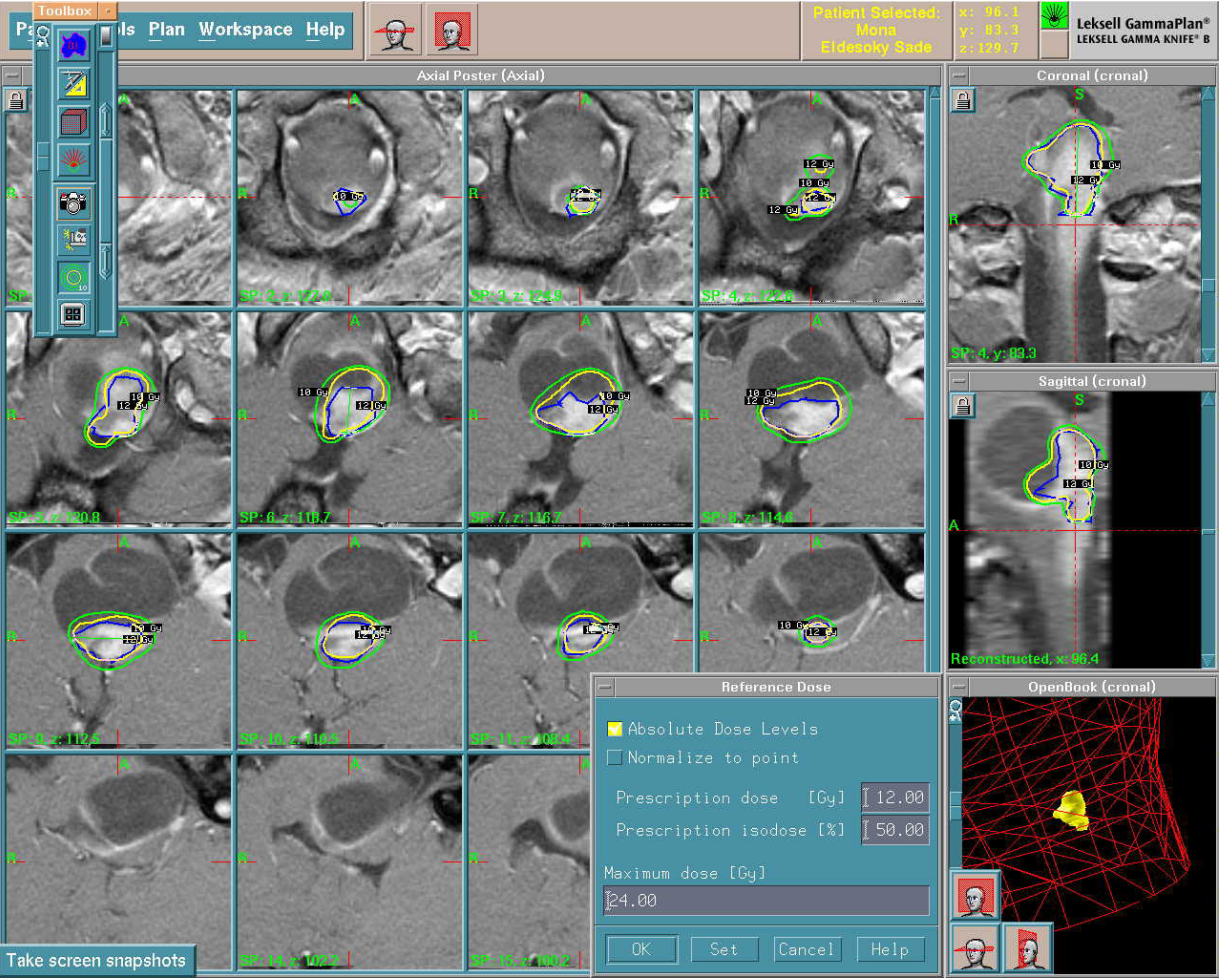
Gamma plan for the treated brain stem recurrent cystic pilocytic astrocytoma.

A 19 year old female patient with partially cystic brain stem (pontine) tumor that was histologically diagnosed as pilocytic astrocytoma, who had undergone two previous surgeries, first an open posterior fossa surgery with partial tumor excision and cyst evacuation in January 2004, and secondly stereotactic evacuation surgery for the cystic part recollection in August 2004 was referred to us on February 2005 with recurrence of her symptoms as repeated infrequent attacks of vomiting, unsteadiness, limb ataxia, and nystagmus. The MRI showed recurrence of the large cystic tumor within the pons, its solid portion extends down to the medulla oblongata (Figure 4&5).

**Figure 6:**
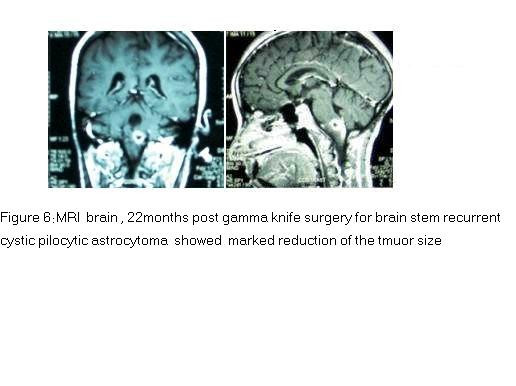
MRI brain, 22 months post gamma knife surgery for brain stem recurrent cystic pilocytic astrocytoma showed marked reduction of the tmuor size.

The patient was treated with gamma knife surgery focusing only to the solid portion of the tumor. The tumor received 12Gy at the margin of the solid portion at 50% isodose curve. At 3 months clinical evaluation showed gradual general improvement. Last MRI done in October 2006 (22 months after GKS) showed marked reduction of the cystic part and even reduced in size of the solid portion (Figure 6).

## Discussion

In our reported cases of recurrent cystic pilocytic astrocytoma which were critically located, gamma knife surgery helped to improve the patient's clinical condition which remained stable for the long time. Radiologically MRI showed progressive reduction of both the solid and almost disappearance the cystic component of the tumor within the follow-up period of, 36 months in the first case with the thalamic lesion and 22 months in the second case with lesion located in the brain stem. Long term follow-up still needed for better and precise evaluation for the GKS role in treatment of pilocytic astrocytoma.

In a study conducted on 19 patients treated at the Karolinska Hospital to evaluate the efficacy of GKS for pilocytic astrocytoma. Sixteen of these patients were children in whom GKS was performed to treat residual tumor after surgery. Most tumors were treated with a prescription dose of 10 to 12 Gy. Tumor control was achieved in all patients. In 85% of the cases a moderate tumor volume reduction was observed after GKS. This result occurred despite the low prescription dose administered [7]

A 7 year old male, right-handed, who presented with a pilocytic astrocytoma in the left parieto-occipital lobe, histologically verified after stereotactic biopsy was treated with Gamma Knife Surgery for recurrence of the tumor cystic portion showed progressive cyst disappearance and mural nodule retraction was obtained during 2 years follow-up. A PET scan performed 3 years after this treatment, revealed no metabolic activity in the persistent retracted mural nodule [9]

Gamma Knife Surgery represents an adjuvant and/or alternative treatment modality for small residual or recurrent low-grade astrocytoma with relatively medium -term local control. GKS minimizes the volume of normal brain tissue irradiation and the possibility of decreasing late side effects of radiotherapy. The treatment of a pilocytic astrocytoma located in a functional area can be performed using GKS [10,11]

## Conclusion

Gamma knife surgery represents an alternate tool in the treatment of recurrent and/or small postoperative residual pilocytic astrocytoma especially if they are critically located minimizes the volume of normal brain tissue irradiation and the possibility of decreasing late side effects of radiotherapy.

## Competing interests

The author(s) declare that they have no competing interests.

## Authors' contributions

**RFH: **conceived and prepared the manuscript.
